# Febrile Urinary Tract Infections in Children: The Role of High Mobility Group Box-1

**DOI:** 10.3390/children10010047

**Published:** 2022-12-26

**Authors:** Roberto Chimenz, Valeria Chirico, Caterina Cuppari, Alessia Sallemi, Davide Cardile, Sergio Baldari, Giorgio Ascenti, Paolo Monardo, Antonio Lacquaniti

**Affiliations:** 1 Pediatric Nephrology and Dialysis Unit, University Hospital “G. Martino”, 98124 Messina, Italy; 2 Nuclear Medicine Unit, University Hospital “G. Martino”, 98124 Messina, Italy; 3 Section of Radiological Sciences, Department of Biomedical Sciences and Morphological and Functional Imaging, University Hospital “G. Martino”, 98124 Messina, Italy; 4 Nephrology and Dialysis Unit, Papardo Hospital, 98158 Messina, Italy

**Keywords:** HMGB1, pyelonephritis, DMSA, renal scar, chronic kidney injury, renal biomarkers

## Abstract

Background: Differentiating between febrile lower urinary tract infection (LUTI) and acute pyelonephritis (APN) is crucial for prompt clinical management. We investigated whether the high mobility group box-1 (HMGB1) could be a useful biomarker in differentiating between LUTI or APN. Methods: We enrolled seventy-four pediatric patients with suspected LUTI/APN, according to the positive or negative renal scintigraphy (DMSA) scan. If the first DMSA findings were abnormal, a second DMSA was performed after six months. Voiding cystourethrography ruled out vesicoureteral reflux (VUR). Results: Higher serum (s) HMGB1 levels characterized the APN group when compared to LUTI patients (13.3 (11.8–14.3) versus 5.9 (5.2–6.8) ng/mL, *p*: 0.02), whereas there were no differences according to urine (u) HMGB1 values. sHMGB1 correlated with C-reactive protein (CRP) levels (β = 0.47; *p*: 0.02). Receiver operating characteristic curves identified the best diagnostic profile for detecting APN. sHMGB1 area under the curve was different from CRP (*p*: 0.01) and white blood cells (*p*: 0.003). After multivariate analyses, VUR (HR:4.81) and sHMGB1 (HR 1.16; *p*: 0.006) were independently associated with the risk of renal scarring development. Conclusions: sHMGB1 could represent a marker to differentiate APN from LUTI. Measurement of sHMGB1 could select children for early intervention or long-term follow-up.

## 1. Introduction

Bacterial urinary tract infection (UTI) commonly involves children, representing a frequent cause of illness. Differentiation between lower and upper UTI is crucial for correct clinical management, underlining that unrecognized and untreated acute pyelonephritis (APN) can lead to renal scarring and potential chronic dysfunction [1]. In addition to the risks during the acute infection, APN can result in long-term deterioration of kidney function, with progression towards chronic kidney disease (CKD) [2,3]. However, the long-term clinical consequences of APN and scar formation on kidney function are controversial, with several studies underlining the low risk of kidney disease and blood hypertension in adulthood after UTI events [4,5].

The current reference standard for renal cortical scar detection after APN is the technetium 99 mTC-dimercaptosuccinic acid (DMSA) isotope nephrogram [6].

After the improved antenatal ultrasound (US) from the 1980s, the diagnosis of congenital anomalies of the kidney and urinary tract (CAKUT) allowed for differentiating APN-related scars, characterized by localized defects, from hypodysplasia, in which kidneys appear uniformly small at US evaluation and with reduced uptake during DMSA scan [7].

Computed tomography (CT) and magnetic resonance imaging (MRI) can also detect APN, but the use of ionizing radiation, radio waves, a magnetic field, and sedation limit their use in clinical practice for young children [8]. Serum and urinary biomarkers have been analyzed for the early detection of APN [9].

Procalcitonin (PCT), rapidly inducted after bacterial stimulation, has been related to the injured renal parenchyma, revealing high diagnostic performance in children with a UTI for the differentiation of APN from lower UTI (LUTI) with high specificity [10].

Neutrophil gelatinase-associated lipocalin (NGAL) predicted early stages of UTI in children and animal models, differentiating bacterial and viral infections [11,12], as well as UTI recurrence in febrile infants and children [13].

However, its low specificity was one of the causes of poor application in clinical practice, considering its variable levels in several unrelated renal diseases, from acute injuries to infective pathologies and chronic kidney disease [14,15].

The host immune response plays a role in uropathogenic *Escherichia coli* (UPEC) infection and scar development. In particular, neutrophils and macrophages are innate immune phagocytes that constitute the first line of defense against bacterial infections. The immune response, elicited during UPEC-induced cystitis, is characterized by rapid infiltration of neutrophils and macrophages in the bladder, leading to infection resolution [16,17].

High mobility group box-1 (HMGB1), belonging to the danger-/damage-associated molecular patterns (DAMPs), also called alarmins, is produced in the early phases by the immune cells, regulating DNA replication, repair, recombination, and transcription [18].

This immuno-stimulatory molecule, which takes part in inflammatory response after tissue injury, triggers innate immunity by activating the NRLP3 inflammasome, G-protein coupled class receptors, the Toll-like receptors, and the receptor for advanced glycation end products (RAGE) [19]. The activation of these receptors promotes several downstream signals, through the protein myeloid differentiation factor 88 (MyD88), inducing inflammation, oxidative stress, as well as the recruitment and activation of neutrophils and macrophages, with the consequent release of HMGB1 from the nucleus to the cytoplasm or even outside the cell, amplifying the inflammation, and activating innate and adaptive immunity [20]. The release of inflammatory cytokines represents the final step of these early and defensive processes, mediated by the nuclear factors κB (NF-κB) activation and translocation and the MAPK and c-Jun N-terminal protein kinase (JNK) pathways [21].

Moreover, in contrast to this immediate activation, occurring after an acute injury, the secretion of HMGB1 level often remains elevated in contexts of chronic inflammation, maintaining and worsening inflammatory disorders, contributing to disease progression through distinct pathways, such as the over-expression of tumor growth factor-beta (TGF-β)-1, resulting in interstitial fibrosis [22].

Furthermore, behind its role in inflammation, HMGB1 exerts a pivotal role in tissue healing and regeneration, acting as a chemoattractant, recruiting stem cells, and regulating angiogenesis [23,24].

HMGB1 has been analyzed in various pediatric infectious diseases, such as enterovirus infections or pneumonia induced by *mycoplasma*; in both cases, high levels of this alarmin characterized these children, correlating with inflammatory cytokines and suggesting its involvement in the inflammatory process [25,26].

Moreover, clinical studies assessed high levels of HMGB1 in pediatric nephropathic patients, revealing high expression in injured renal tissues [27,28].

In the present study, we investigated whether HMGB1 could be a useful biomarker in differentiating between children with a clinical suspicion of febrile LUTI or APN, considering that, to date, no data are available. 

## 2. Patients and Methods 

### 2.1. Study Design

The Pediatric Nephrology and Dialysis Unit, University Hospital “G. Martino” Messina, Italy, conducted the enrollment from January 2018 to June 2021, enrolling seventy-four febrile pediatric patients with suspected UTI/APN.

Inclusion criteria: fever ≥ 38.5 °C, pyuria defined as ≥10 white blood cells per high-power field on centrifuged urine, and positive urine culture, defined as the growth of one organism at ≥104 CFU/mL from a catheterized specimen or ≥105 CFU/mL from a clean catch, midstream, or bag specimen.

Exclusion criteria: urologic or anorectal malformations, single kidney, concomitant active infective diseases, such as pulmonary or gastrointestinal infections, inflammatory disorders, neurologic disorders, immunodeficiency, antibiotic treatment administered in the past six months, and urological intervention within the last six months. Septic neonates, often characterized by hypothermia or absence of fever, were excluded from the enrollment. We excluded participants with chronic kidney injury or proteinuria, which were not associated with concomitant APN. The creatinine-based “bedside Schwartz” equation calculated the estimated glomerular filtration rate (eGFR) [29].

**Figure 1:**
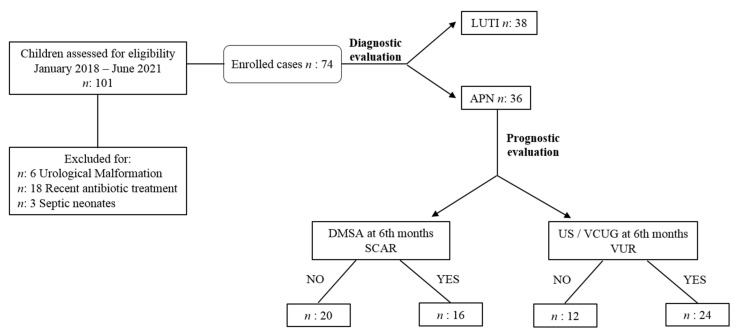
Study design. Abbreviations: LUTI: lower urinary tract infection; APN: APN: acute pyelonephritis; DMSA: technetium 99 mTC-dimercaptosuccinic acid renal scintigraphy; US/VCUG: ultrasound/ voiding cystourethrography; VUR: vesicoureteral reflux.

We hospitalized all children aged <2 years or if they needed a parenteral infusion of antibiotics. All the other patients were treated at home or during a day-hospital regimen. A positive DMSA renal scintigraphy defined APN [30]. In particular, after the admission with UTI, a DMSA scan was performed in all enrolled patients within the first ten days, considering that the accuracy of this test decreases if obtained later than two weeks after the diagnosis of UTI [31]. If the DMSA scan revealed perfusional defects, patients belonged to the APN group, whereas negative DMSA characterized LUTI patients. Figure 1 summarizes the study design.

Before the initiation of antibiotic treatment, we sampled blood for laboratory investigations. We included only patients who had received blood sampling before antibiotic administration because the concomitant antibiotic therapy could affect HMGB1 levels.

We enrolled twenty healthy subjects (HS group), well matched with patients for age and gender, including only children whose routine urinalysis and urinary albumin creatinine ratio were normal.

The Ethics Committee of the University of Messina approved the study (protocol Number 120/19). Both parents of each patient provided written informed consent.

### 2.2. Diagnostic Tests

DMSA scans were performed with a gamma camera after the intravenous injection of a dose of technetium-99 DMSA with weight adjustment. Single-photon emission computed tomography was performed for four hours after the isotope administration for a scan time of 20 min. The current reference standard for assessing the presence and extent of pyelonephritis is to conduct a planar DMSA renal scan. When radiolabeled DMSA is given to patients with impaired tubular cells due to APN, the scan will show a photon-deficient area(s). Trained nuclear medicine physicians conducted the test in a hospital’s radiology department. For this analysis, any photopenia (with or without a loss of contours) identified pyelonephritis.

### 2.3. HMGB1 Analysis

HMBG1 levels were measured through HMGB1 ELISA kit II (IBL by Shino-test corporation, Japan). The detection limit is 0.2 ng/mL +2.6 SD for the high sensitivity range; the limit of quantification is 0.1 ng/mL with a coefficient of variation (CV) ≤ 20% for higher sensitivity. The samples have been diluted 1:5 in diluents, added to microplates, and then incubated for 24 h at 37 °C. After washing, 100 micro/L of anti-HMGB1 antibodies and monoclonal antibodies conjugated have been added and incubated at room temperature for two hours. A standard curve with a 0.2 ng/mL minimum detection limit deducted HMGB1 concentrations. 

### 2.4. Follow-Up Period

After the baseline assessments, if the first DMSA findings were abnormal, another analysis was performed after six months to assess irreversible renal damage. 

According to this second DMSA analysis, pathological proteinuria, associated or not with an eGFR reduction, defined the patient as affected by chronic kidney dysfunction. Patients were contacted if they missed an appointment, avoiding data loss during the follow-up. 

Moreover, children with renal ultrasound alterations underwent voiding cystourethrography (VCUG) to rule out vesicoureteral reflux (VUR) [32,33].

### 2.5. Statistical Analyses

Statistical analyses were performed using the SPSS for Windows version 18.0 (SPSS Inc., Chicago, IL, USA) and GraphPad Prism software (GraphPad Software. 2365 Northside Dr. Suite 560 San Diego, CA 92108, USA). Data were presented as mean ± SD for normally distributed values (at Kolmogorov–Smirnov test) and median (IQ range) for non-normally distributed values. Differences between groups were established by an unpaired *t*-test, for normally distributed values and by Kruskal–Wallis analysis followed by Dunn’s test for nonparametric values.

The Pearson and the Spearman correlation coefficients were employed to test correlations between variables, as indicated. Moreover, a point-biserial correlation was performed to evaluate associations between HMGB1 and dichotomous variables, such as gender. Before testing correlations, all values showing a skewed distribution were log-transformed to better approximate normal distributions. Multiple regression analyses were performed by building a model including all univariate correlates of HMGB1 to assess independent relationships. Data were expressed as partial correlation coefficients (β) and *p*-value.

Receiver operating characteristics (ROC) analysis calculated the area under the curve (AUC) for white blood cells (WBC), hsCRP, and sHMGB1 to find the best cut-off values for the predictive value for diagnosing APN. Adjusted risk estimates for renal scar in APN patients were calculated using Cox proportional hazard regression analyses. All results were considered significant if *p* was <0.05.

## 3. Results

### 3.1. Patients’ Baseline Characteristics

Table 1 summarizes the main baseline characteristics of the study cohort.

The study group included 74 children, 36 female (49%) and 38 male (51%), and median (IQ range) age at presentation was 3 (1–6.5) years. Mean serum creatinine was 0.6 ± 0.29 mg/dL, with a mean eGFR of 106.7 ± 12.40 mL/min, whereas albuminuria and proteinuria were in the normal range at the enrollment phase. 

APN was revealed in 36 patients (48%), whereas 38 patients had a LUTI, with a median (IQ range) age of 2 (1–5) and 7 (4–10.5) years, respectively. The mean WBC count was significantly higher in the APN group (16.756 ± 3.174 cells/mm^3^) than in the LUTI group (8.168 ± 2.256 cells/mm^3^; *p* < 0.0001), as well as hsCRP [18.5 (12–28) vs. 2.1 (1.3–4) mg/dL; *p* < 0.0001]. We detected *Escherichia coli* in forty-nine (66%) patients, whereas the rest of the patients had other urinary microbial profiles, such as *Klebsiella pneumoniae* (*n*: 12, 16%), *Pseudomonas aeruginosa* (*n*: 8, 11%), and *Eterococci* (*n*: 5, 7%). 

### 3.2. HMGB1 Levels and Correlations 

**Figure 2:**
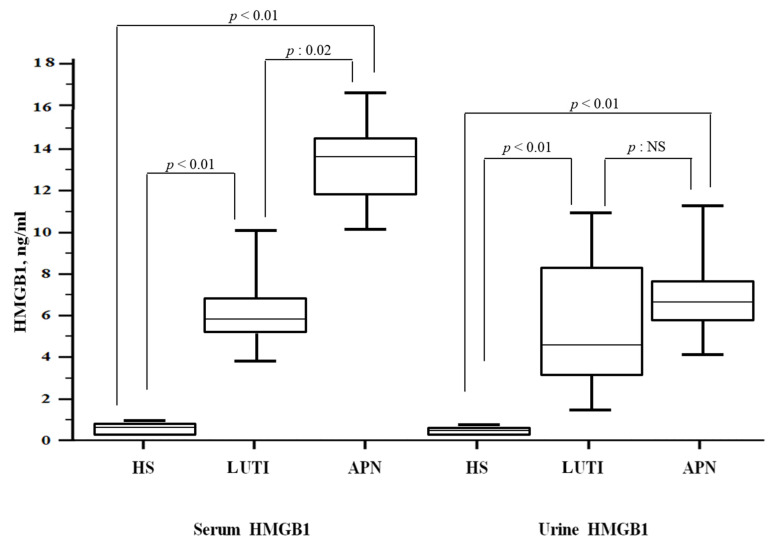
HMGB1 levels in the study population. Abbreviations: HMGB1: high mobility group box 1; LUTI: lower urinary tract infection; APN: acute pyelonephritis; HS: healthy subjects.

Higher serum HMGB1 levels characterized the APN group when compared to LUTI patients [13.3 (IQR 11.8–14.3) vs. 5.9 (IQR 5.2–6.8) ng/mL; *p*: 0.02], whereas there were no differences between the two groups according to uHMGB1 values [6.2 (IQR 5.7–7.4) vs. 4.3 (IQR 3–8.2) ng/mL; *p* = 0.12]. When compared to HS (sHMGB1: 0.6 (IQR 0.5–0.7) ng/mL; uHMGB1: 0.6 (IQR 0.4–0.7) ng/mL), LUTI and APN patients had higher levels of both serum and urine HMGB1 values (Figure 2).

At univariate analysis, logsHMGB1 directly correlated with loghsCRP (R = 0.41; *p*: 0.0004), WBC (R = 0.36; *p*: 0.002), and platelet count (R = 0.33; *p*: 0.008). In contrast, no correlation was found for other parameters such as age, body mass index (BMI), hemoglobin, creatinine levels (R range from 0.06 to 0.18; *p*: > 0.06), and uHMGB1 levels. 

Moreover, HMGB1 did not correlate with gender, according to a point-biserial correlation.

All variables, significantly correlated with sHMGB1 at univariate analysis, were introduced in a multivariate model using sHMGB1 as a dependent variable. After adjustment for other factors, significance was maintained for the correlation between HMGB1 and hsCRP (β = 0.47; *p*: 0.02). In contrast, the correlations with WBC and platelet count found in a univariate analysis were lost. This multivariate model explained about 62% of the total variance of HMGB1 in this cohort. Table 2 depicts findings at univariate and multiple regression analysis.

### 3.3. ROC Curves

**Figure 3:**
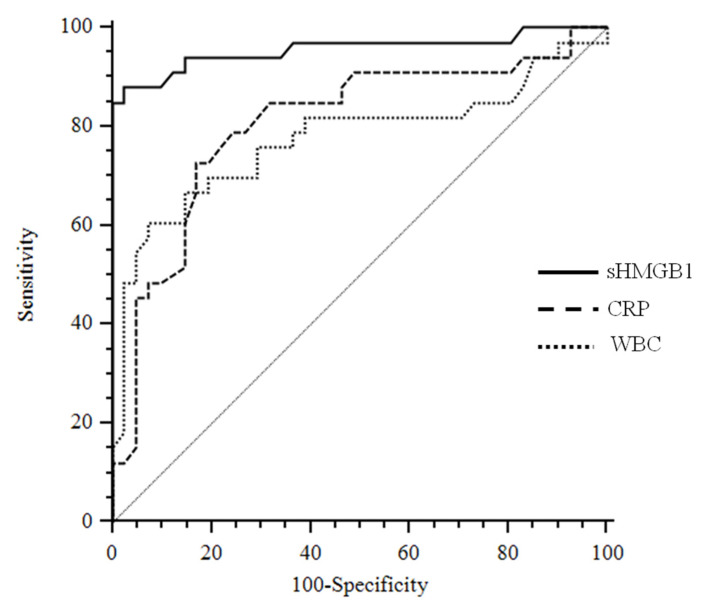
Receiver operating characteristics curves sHMGB1 considering APN as a status variable. Abbreviations: sHMGB1: serum high mobility group box 1; CRP: C reactive protein; WBC: white blood cells.

ROC analyses determined the best cut-off values of sHMGB1, hsCRP, and WBC for identifying APN in the cohort group (Figure 3).

The area under the curve (AUC) values for sHMGB1 (AUC: 0.956; 95% CI: 0.881–0.989) were significantly different from hsCRP (AUC: 0.805, 95% CI: 0.696–0.888; *p*: 0.01) and WBC (AUC: 0.775; 95% CI: 0.663–0.864; *p*: 0.003). Similar results were obtained if the WBC and hsCRP were combined together and compared to HMGB1 curve. On the contrary, the difference between the hsCRP and WBC AUC was not significant (*p*: 0.56). 

The best cut-off value of sHMGB1 to predict APN was >9.1 ng/mL, with a sensitivity of 87.9 (95% CI, 71.8 to 96.6) and a specificity of 97.6 (95% CI, 87.1 to 99.9), whereas for hsCRP was >9.6 mg/dL with a sensitivity of 72.7 (95% CI, 54.5–86.7) and a specificity of 82.9 (95% CI, 67.9 to 92.8). 

### 3.4. Follow-Up Period

A second DMSA was performed in thirty-six APN children six months later, and all patients with abnormalities detected by ultrasound (US) evaluation underwent VCUG to rule out a VUR.

The scars persisted in 16 children (44%), with remission of the renal lesion in 20 patients (56%), albuminuria and proteinuria levels remained within normal ranges, as well as serum creatinine, in all 36 patients. Kidney US revealed abnormalities in 24/36 patients (67%) who underwent VCUG. In particular, severe hydronephrosis and associated VUR (grades 3–5) characterized patients with persistent scars (Table 3).

### 3.5. Univariate/Multiple Cox Regression Analysis and Persistent Renal Scar

To identify putative risk factors associated with persistent renal scarring, we performed a Cox regression analysis, inserting in the model all variables that were different at baseline in patients who had renal scars at DMSA evaluation after the follow-up period (hydronephrosis, VUR, sHMGB1, hsCRP, and WBC). In univariate and multivariate analysis, VUR (HR 4.81; 95% CI, 1.41 to 16. 3; *p*: 0.01) and sHMGB1 (HR 1.16; 95% CI, 1.04 to 1.29; *p*: 0.006) were independently associated with the development of renal scarring.

## 4. Discussion 

sHMGB1 represents a diagnostic biomarker in APN children characterized by higher levels than the LUTI and HS groups. sHMGB1 values > 9.1 ng/mL as a cut-off level, ten times higher than values observed in healthy children, which could identify children with APN.

An amount of 99 mTc-DMSA scintigraphy is a commonly adopted method, but several issues limit its employment in clinical practice, such as the placement of an intravenous line, sedation requirement, and radiological exposure. Its specificity is not absolute, not differentiating old scars due to relapsing pyelonephritis or acute kidney injuries [34].

We also found a positive correlation between sHMGB1 and hsCRP. The latter, such as WBC and PCT, could be more helpful for ruling out APN if low levels occur, and according to a recent Cochrane review, none of these tests was accurate enough to differentiate upper from lower urinary tract disease [35].

Children with APN are vulnerable to renal scarring due to the necrosis and fibrosis associated with acute systemic inflammation. The release of various mediators, including HMGB1, amplifies the clinical expression of APN, with systemic involvement not only restricted to the kidneys. Conversely, in LUTI, cytokines are secreted mainly in the urine, without a systemic disease [36].

**Figure 4:**
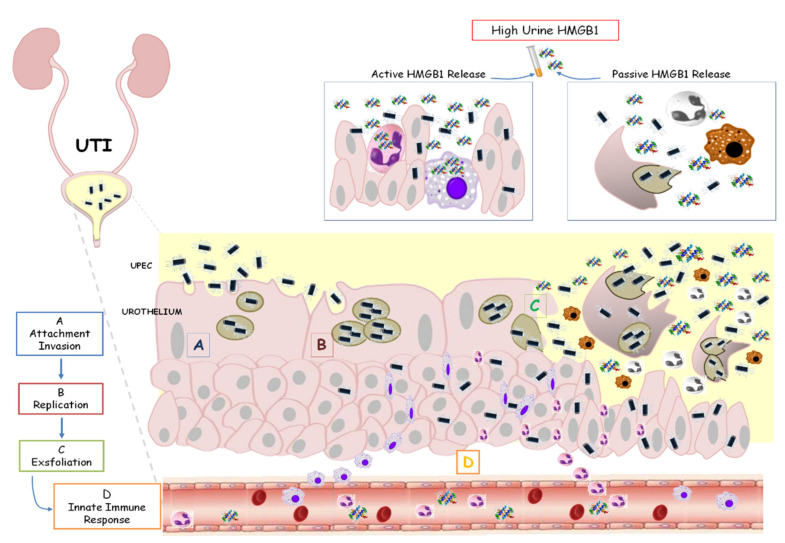
HMGB1 involvement in urinary tract infection. Uropathogenic *Escherichia coli* (UPEC) binds and invades the urothelium (**A**,**B**). Host inflammatory responses begin to clear extracellular bacteria. However, some bacteria evade the immune system, producing toxins and proteases that induce host cell damage with a final cell exfoliation (**C**). The activation of the innate immune system is based on the secretion of chemical mediators, such as chemokines and cytokines (**D**). In this contest, HMGB1 is passively released into urine from damaged or necrotic cells or actively released from activated cells, such as inflammatory cells and immune cells. High urine levels of HMGB1 characterized children with lower UTI.

This analysis supports our data about high uHMGB1 levels assessed only in LUTI patients as an expression of limited urinary infection, without a systemic impairment, as occurred during APN (Figure 4).

Predicting high-risk patients and selecting appropriate diagnostic tools is crucial to treat children suspected of genitourinary injury. We demonstrated that high levels of sHMGB1, associated with VUR, were independently associated with an increased risk of APN. The persistent immune system activation and systemic inflammation could contribute to the pathogenesis of structural kidney damage behind morphologic alterations related to VUR. Dysfunctional voiding or VUR predisposes to APN, inducing an increased frequency of UTI mediated by bacteria of lower virulence [37]. Moreover, the host response to the presence of bacteria in the urinary tract might be genetically determined, suggesting that genetic polymorphisms of cytokines may influence the type of host response during an infection. Low cytokine responders may not have systemic symptoms despite bacteria in their urine [38].

Previous research in experimental APN has uncovered that Toll-like receptor-4–/–, interleukin-1β–/–, or mice treated with forskolin (which has anti-inflammatory properties) had attenuated tissue inflammation and less severe acute kidney infection, suggesting that excessive inflammatory responses could be harmful instead of beneficial for the host [39,40].

**Figure 5:**
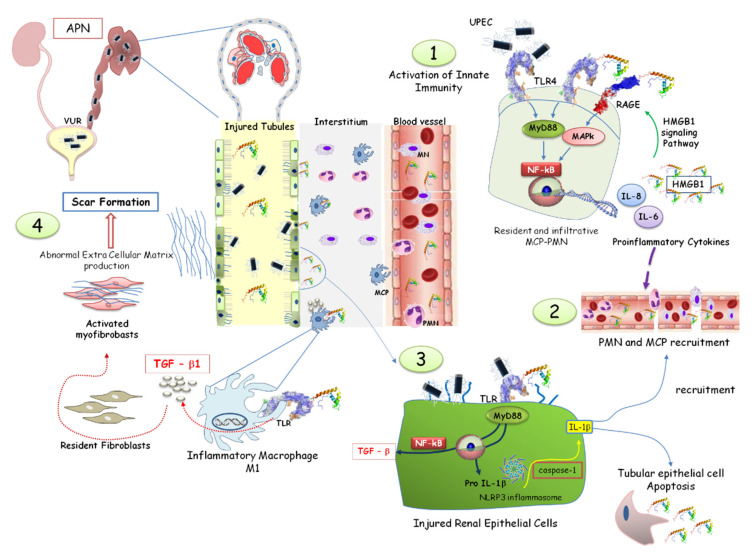
HMGB1 involvement in acute pyelonephritis. Neutrophils (PMN) and macrophages (MCP) are activated by HMGB1, recognized by the toll-like receptor (TLR), leading to the activation of the NF-system and the synthesis of pro-inflammatory proteins (**1**,**2**). HMGB1-mediated pathways promote the assembly of the NLRP3 inflammasome, amplifying the inflammatory response and immune cell recruitment. HMGB1 promotes TGF-*β1* synthesis by renal epithelial cells through the NF-*κB* pathway (**3**). In the interstitial renal tissue, activated monocytes differentiate into M1 macrophages, producing TGF-β. The consequent myofibroblast activation, proliferation, and extracellular matrix deposition induced a vicious circle increasing HMGB1 and TGF-β synthesis. Renal fibrosis and scar formation represented the final process (**4**).

In this context, the over-expression of HMGB1 represents a continuous stimulation of Toll-like receptors, inducing a tubule-interstitial inflammatory response via the NF-κB signaling pathway, which chronically leads to the over-expression of tumor growth factor-beta (TGF-β)-1. That is the starting point for amplified immuno-stimulatory and inflammatory responses resulting in interstitial fibrosis and renal scarring [41,42] (Figure 5).

Similar processes involved chronic nephropathic children, such as Alport syndrome or lupus nephritis. Different nephrotoxic mechanisms involving HMGB1 pathways had a common final effect: fibrosis [27].

HMGB1 directly induces the interstitial accumulation of macrophages, promoting their differentiation to the M1 phenotype, which is critical for the onset of interstitial fibrosis [43,44]. Moreover, this alarmin might be a marker of acute inflammation and renal structural impairment even without acute kidney injury, as revealed by serum creatinine, GFR, and proteinuria within normal ranges assessed in our patients. sHMGB1 levels did not correlate to these functional renal markers.

The complexity of these processes, mediated and induced by this alarmin, probably explains why HMGB1 should not be defined as a simple inflammatory peptide, acting only as a consequence of an infective trigger, as observed during APN. Its diagnostic properties were superior to those obtained through hsCRP and WBC evaluation.

Improved knowledge about APN pathogenesis and renal scarring will lead to effective strategies, reducing recurrent LUTI and renal fibrosis [45].

We demonstrated the critical impact that APN could have on kidney structure and function. Six months after the acute infection period, 16 out of 36 patients (44%) had confirmed renal scarring. However, further studies would better analyze the role of HMGB1 from a prognostic point of view, considering that the cohort of patients was small to draw a significant conclusion. Moreover, rapid tests should be necessary, to obtain immediate information and actions, such as antibiotic start or precocious DMSA procedure. The localization of infection can be considered a first step in the UTI investigation, and the DMSA renal scintigram could represent the test of choice for the diagnosis of APN, given the low specificity of clinical findings and available laboratory tests [46,47,48,49].

Neither classical clinical and biochemical signs and symptoms nor ultrasound distinguished between a true APN and febrile infections not involving the kidney [50]. However, false negative results of the DMSA test cannot completely rule out APN [51,52], especially in children aged <2 years.

## 5. Conclusions

We first assessed the role of HMGB1 in the APN and LUTI pediatric population.

sHMGB1 could represent a marker to identify children with UTI, differentiating APN from LUTI. The increase of HMGB1 may express the degree of subclinical renal impairment representing an earlier measurable index of renal suffering, compared with classic glomerular and tubular signs, such as proteinuria or eGFR. Multicenter, prospective studies should evaluate if sHMGB1 could be a reliable diagnostic marker for APN in UTI pediatric patients associated with other inflammatory cytokines and interleukins.

## Figures and Tables

**Table 1:** Clinical and Biochemical Parameters of Patients with Acute Pyelonephritis and Lower Urinary Tract Infection at Baseline.

Parameter	All Patients (*n*: 74)	APN Group (*n*: 36)	LUTI Group (*n*: 38)
Median age, years	3 (1–6.5)	2 (1–5)	7 (4–10.5)
M/F	36/38	21/15	15/23
BMI, Kg/m^2^	18.2 ± 3.7	16.6 ± 2.1	18.6 ± 2.7
WBC count (×10^3^)	12.3 ± 5.1	16.7 ± 3.1	8.1 ± 2.2
hsCRP, mg/dL	6.6 (2–16.7)	18.5 (12–28)	2.1 (1.3–4)
sCreatinine, mg/dL	0.6 ± 0.2	0.5 ± 0.3	0.6 ± 0.1
eGFR, mL/min	106.7 ± 12.4	103.2 ± 9.1	105.7 ± 5.6
sHMGB1, ng/mL	6.3 (5.1–14.4)	13.3 (11.8–14.3)	5.9 (5.2–6.8)
uHMGB1, ng/mL	6.1 (2.9–8.5)	6.2 (5.7–7.4)	4.3 (3–8.2)
Positive 1st DMSA *n*	36	36	0

Abbreviations: APN: acute pyelonephritis; LUTI: lower urinary tract infection; BMI: body mass index; WBC: white blood cells; hsCRP: high sensitivity C reactive protein; sCreatinine: serum creatinine; eGFR: estimated glomerular filtration rate; sHMGB1: serum high mobility group box 1; uHMGB1: urine high mobility group box 1; DMSA: technetium 99 mTC-dimercaptosuccinic acid renal scintigraphy.

**Table 2:** Univariate and multiple regression analysis of (log-transformed) sHMGB1 levels.

	Univariate Correlation Coefficient	*p*	Multivariate Correlation Coefficient (β)	*p*
(log) hsCRP	0.41	0.0004	0.47	0.02
(log) uHMGB1	0.27	0.10		
(log) Age	0.12	0.16		
BMI	−0.21	0.10		
Hemoglobin	0.16	0.21		
Platelet Count	0.33	0.008	0.10	0.14
Creatinine	−0.23	0.09		
WBC count	0.36	0.002	0.39	0.08

Statistical significance is highlighted in bold. Multiple R = 0.75, R^2^ = 62%; *p* < 0.01. Abbreviations: hsCRP: high sensitivity C reactive protein; uHMGB1: urine high mobility group box 1; BMI: body mass index; WBC: white blood cells; sHMGB1: serum high mobility group box 1.

**Table 3:** Patients with Acute Pyelonephritis after the follow-up period.

Follow-Up Period (Sixth Month)		
APN Group (*n*: 36)	Persistent Scars *n*: 16 (44%)	No Scars *n*: 20 (56%)
I-II grade Hydronephrosis, *n*	1	9
III-IV grade Hydronephrosis, *n*	13	1
VUR, *n*	14	10
I-II grade VUR, *n*	1	9
III-IV grade VUR, *n*	13	1
hsCRP, mg/dL	23.7 (17.3–27.4)	13.5 (9.7–21.2)
WBC count (×10^3^)	17.7 ± 2.7	13.8 ± 2.2
sHMGB1	13.9 (12.2–16.8)	10.7 (8.9–11)

Abbreviations: APN: acute pyelonephritis; WBC: white blood cells; hsCRP: high sensitivity C reactive protein; sHMGB1: serum high mobility group box 1; VUR: Vesicoureteral Reflux.

## Data Availability

The dataset generated and analyzed during the current study is available from the corresponding author on reasonable request.
